# Attempts to standardize intradermal drug tests based on molecular mass and on clinical phenotypes. Some pitfalls or exceptions?

**DOI:** 10.1186/2045-7022-4-S3-P102

**Published:** 2014-07-18

**Authors:** Joseph Mathias Baló Banga, Adrienne Vajda

**Affiliations:** 1 Dermato-Allergy Unit, Department of Dermatology Medical, Hungary; 2 Dermato-Allergy Unit, Department of Dermatology Medical, Medical Center of the Hungarian Defense Forces, Hungary

## Background

Despite many efforts to standardize drug skin tests only a few position papers appeared. They were based mainly on prick tests and to lesser extent on intradermal testing.Their standardization is a task set by an ENDA group. Our aim was to re-evaluate our drug skin tests (except patch testing) performed during the past 30 years in a retrospective study and to compare it with a newer protocol applied since 2009.

## Methods

In the first phase 185 "unselected" patients have been tested upon suspicion of drug hypersensitivity causing skin or general symptoms. Drug solutions of 1x10-3M against solvent were injected into the volar surfaces of forearms (403 tests). In 17 cases parallel tests with scarification and 81 oral provocations with respective drugs have been carried out as well. Tests were red at 20'; 70-90' and 24 hrs. Drugs: Penicillin G, V; Ampicillin; Aminopyrin; Sulfadimidin; Sulfametoxazol; Phenobarbital; Acetylsalicylic acid. The clinical phenotypes of the hypersensitive event fell into 15 categories: including MPE (26%), localized urticaria with/without ANO (19%), generalized urticaria (19%) erythema multiforme, TEN (4% each), Stevens Johnson's and anaphylaxis (3.4% each) and others. Positivity was accepted simply on the two fold increase of the reaction against controls irrespective of its character: wheal or erythema. The "improved" test (2009-) included additionally 5-10 µg/ml histamine as positive control and restriction of positivity only to wheal >5mm. Suspect patients (26) and 20 controls were tested. The phenotypes due to previous experience were MPE, Urticaria, ANO, AD, Anaphylaxis, disseminated Erythemas.

## Results

In the first phase out of 205 intradermal tests positivity has occurred at 20' in 62%, at 70-90' in 52% and at 24 hrs. in 33%. Scarification was all negative. 42% of oral provocations were positive. An indication for intradermal tests based on the clinical phenotype was set up: MPE >45% (body surface), Urticaria ±ANO (10-30%), Erythema multiforme, Bullous lesions of small extension, Anaphylaxis (gr.I-II). All other phenotypes should not be tested. Comparing "unselected" with "selected" group and testing various drugs against provocation the overall intradermal positivity went up from 43.2% to 77.5 %. "Unselected" negativity was 94% against "selected" one of 80%. Histamine as positive control has caused 103 mm wheal with 20-50 mm red halo. Drugs with mol. mass <1000 Da were suitable for standard test.

